# Gut microbiota of the European Brown Hare (*Lepus europaeus*)

**DOI:** 10.1038/s41598-019-39638-9

**Published:** 2019-02-25

**Authors:** G. L. Stalder, B. Pinior, B. Zwirzitz, I. Loncaric, D. Jakupović, S. G. Vetter, S. Smith, A. Posautz, F. Hoelzl, M. Wagner, D. Hoffmann, A. Kübber-Heiss, E. Mann

**Affiliations:** 1 Research Institute of Wildlife Ecology, Department of Integrative Biology and Evolution, University of Veterinary Medicine, Vienna, 1160 Vienna Austria; 2 Department for Farm Animals and Veterinary Public Health, Institute for Veterinary Public Health, University of Veterinary Medicine, Vienna, 1210 Vienna Austria; 3 Department for Farm Animals and Veterinary Public Health, Institute of Milk Hygiene, Milk Technology and Food Science, University of Veterinary Medicine, Vienna, 1210 Vienna Austria; 4 Austrian Competence Centre for Feed and Food Quality, Safety and Innovation FFoQSI GmbH, Technopark 1C, 3430 Tulln, Austria; 5 Department of Pathobiology, Institute of Microbiology, University of Veterinary Medicine, Vienna, 1210 Vienna Austria; 6 Konrad Lorenz Institute of Ethology, Department of Integrative Biology and Evolution, University of Veterinary Medicine, Vienna, 1160 Vienna Austria; 7 Game Conservancy Deutschland e. V., Schloßstrasse 1, 86732 Oettingen, Germany

## Abstract

Diseases of the gastrointestinal tract due to changes in the bacterial flora have been described with increasing incidence in the European brown hare. Despite extensive demographic and phylogeographic research, little is known about the composition of its gut microbiota and how it might vary based on potential environmental or host factors. We analysed the intestinal and faecal microbiota of 3 hare populations by Illumina MiSeq 16S rRNA gene amplicon sequencing. The phyla and OTU abundance composition differed significantly between intestinal and faecal samples (PERMANOVA: *P* = 0.002 and *P* = 0.031, respectively), but in both sample types *Firmicutes* and *Bacteroidetes* dominated the microbial community composition (45.51% and 19.30% relative abundance). Intestinal samples contained an enrichment of *Proteobacteria* compared with faecal samples (15.71-fold change, *P* < 0.001). At OTU level, a significant enrichment with best BLAST hits to the *Escherichia-Shigella* group, *Eubacterium limosum*, *Sphingomonas kyeonggiensis*, *Flintibacter butyricus* and *Blautia faecis* were detected in intestinal samples (*P* < 0.05). In our statistical model, geographic location and possibly associated environmental factors had a greater impact on the microbiota composition than host factors. Population had a significant effect on the composition of abundant intestinal and faecal OTUs, and on the abundance of potential pathogenic bacteria of the family *Enterobacteriaceae*, regularly associated with intestinal dysbiosis in hares, in faecal samples. Our study is the first to describe the microbiota in brown hares and provides a foundation to generate hypothesis aiming to test the role of gut health in population fluctuations of the species.

## Introduction

The European brown hare (EBH) (*Lepus europaeus*) is a widely distributed and important game species throughout Europe. Despite the classification of their population status as “least concern” by the International Union for Conservation of Nature (IUCN, red list 2017), significant population declines have been noted in the last 50–60 years leading to a “near threatened” or “threatened” status on national red lists for some regional populations, e.g.^1^. A range of factors including unfavourable climatic conditions^2^; predation^3^ and multiple disease epidemics such as the European brown hare syndrome (EBHS), Pasteurellosis, Pseudotuberculosis or Coccidiosis^4–6^ have contributed to regional threats. However, probably the most important threat for this species is the intensification of agriculture^7–10^. Habitat fragmentation, agricultural mechanization, monocultures, unpredictable food availability as well as pesticide and fertilizer use have been shown to have detrimental health effects on a variety of farmland wildlife species^11–13^ including substantial impact on hare population viability^7,8^. Species like the brown hare can be even more severely affected as the species’ diet is highly selective and adapted to maximise energy intake by positively selecting certain plants species^14^. Furthermore, lagomorphs are known to be very sensitive to any feed alterations and imbalances, causing disruption of the gut microbiota and resulting gastrointestinal disease^15–18^. The pathology is associated with an intestinal bacterial dysbiosis, which might lead to an increase of pathogenic bacteria such as e.g. *Escherichia coli* or *Clostridium* spp. and often results in reduced fitness, enteritis and other diseases or even death^19,20^. Results of recent health screenings of hare populations showed such changes in the gut microbiota^21^.

Recent research in human and veterinary science has highlighted the importance of the gut microbiota impact on host physiology^22–25^. A variety of host specific factors, such as age, sex, body condition, genetics and host phylogeny are known to shape the microbiota in an individual^26–30^. Additionally, the role of environmental factors, such as lifestyle, diet, climate and habitat conditions, as well as changes in land use are described to impact the microbial composition in the gastrointestinal tract (GIT)^31–33^. Nevertheless, this knowledge cannot be easily transferred to wildlife species. Wild animals face highly diverse and changing environments. They have established physiological plasticity and acclimatization mechanisms that differ greatly from domestic or laboratory animals^34^.

Generating physiological baseline data of populations living in their natural habitats is important in order to understand and predict what factors might have the most impact on a species. This requires studies of wildlife species within their natural habitats^33,34^. So far, studies investigating the gut microbiota of free-ranging wildlife are rare and baseline data of potentially influencing factors of microbial community composition, abundance of specific bacterial taxa associated with host health in wildlife species is lacking^30,33,35,36^. Studies comparing wild with captive wildlife populations have reported differing gut microbiota compositions associated with a highly co-evolutionary adapted microbiota in the investigated wildlife species and a loss of diversity in captive animals^37^. The importance of the gut microbiome has recently also been more and more considered in species conservation and management measures^38,39^.

In this study, we analysed the gut bacterial microbiota composition in European brown hares with 16S rRNA gene sequencing and assessed differences between intestinal and faecal samples. By the use of these molecular high-throughput techniques and community analysis we aimed to identify and describe the gut microbiota, a goal that could not be achieved by previous culture- and/or enrichment-dependent methods.

Furthermore we used a statistical model to investigate possible impacts of host factors, sampling population and potentially associated different land use on the gut microbiota composition of free-ranging European brown hare. Specifically, we tested the impact of host factors such as age, sex, body condition (heart fat) and gut health as well as sampling population on the abundance and diversity of the bacterial composition in intestinal and faecal samples of brown hares.

## Material and Methods

### Sample and data collection

**Figure 1:**
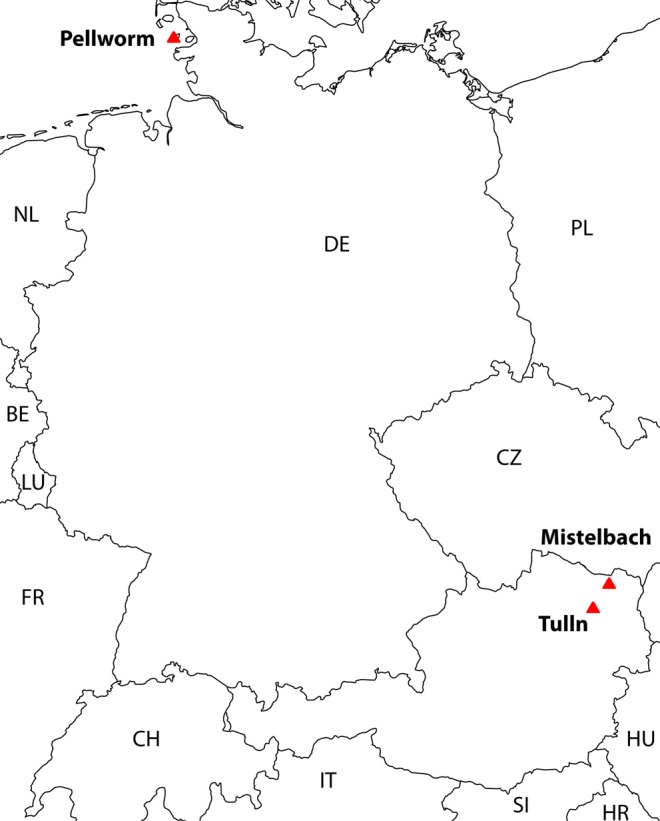
Sampling locations of European brown hares (labelled with red triangles): Sampling area (**a**) Pellworm, Germany (Grassland type) is characterized by 70% grassland and 30% cropland of the total agricultural area; (**b**) Lower Austria, district Mistelbach, Austria (Cropland-type) by 79% of the agricultural area being used as cropland and 0.4% as grassland **(c)** Military Airport Langenlebarn; Tulln, Austria, (no agricultural use). For a detailed description see also Material and Methods.

A total of 25 brown hares (*Lepus europaeus*) of both sexes were included in this study. Metadata of all animals included is listed in Supplementary Table S1. The study including the experimental protocol was carried out and approved in accordance with all current laws of Austria and the guidelines and regulations of the institutional Ethics Committee and the institutional Good Scientific Practice Guidelines of the University of Veterinary Medicine, Vienna. Samples were all obtained during the hunting season in December 2015, in order to exclude any seasonal effects. In all hunting, respectively sampling areas, a systematized yearly drive-hunt using rifles according to the respective hunting law was performed. Sampling locations are spatially visualized in Fig. 1. The two Austrian populations are known to genetically diverse with geneflow occurring both locally and regionally^40,41^. The population on Pellworm has more restricted genetic diversity typical of an isolated island population (unpublished data). Sampled individuals were from well separated areas within each hunting ground to avoid the possibility of including individuals from the same family groups within each population sample.

As age could constitute an influential factor on the microbiota of hares, the age was determined by the weight of the dried eye lenses^42^. This method allows for the sensitive discrimination of subadult hares (i.e. young of the year or individuals not older than 9–10 months of age) that can otherwise not easily be distinguished from adult hares based on morphological characteristics. Hares that were younger than one year of age were classified as subadults. Digesta samples from the cranial flexure of the duodenum (intestinal samples) and faecal samples from the *ampulla recti* were collected in sterile sampling vials during a standardized necropsy by trained pathologists in the field within 20 minutes after death and immediately frozen at −20 °C, transported to the lab and frozen at −80 °C until use.

Heart fat (coronary fat deposit) as a parameter defining the nutritional status was assessed ranging from 1–6, such that 1 defined cachectic, 2 bad, 3 moderate, 4 good, 5 very good and 6 described obese. The parameter gut health was defined based on the following variables assessed by pathological examination of the gastrointestinal tract: 1: no macroscopic and/or no/very mild histopathological lesions (smooth mucosa, no signs of inflammation (0–+), no increase in inflammatory cells); 2: moderate dysbiosis signs of inflammation, ((+–++) inflammatory cells); 3: severe macroscopic and/or histopathological lesions (severe mucosal erythema, severe signs of inflammation, and (++–+++) inflammatory cells). The animals originated from three different geographical locations associated with different land use practices. As hares are highly selective feeders that prefer weeds/grasses and various crop types while avoiding cereals^14^, we chose the sampling sites according to the different land use types: (a) Grassland-type (Pellworm): Around 80% of the whole area of the island, Pellworm, northern Germany, are used as agricultural land; around 70% of the agricultural land consists of grassland (pastureland and green fodder for domestic species such as cattle and sheep); the remaining 30% are used as cropland; (main crop consists of winter wheat (*Triticum aestivum*), rapeseed (*Brassica napus*), as well as to a lower percentage, barley (*Hordeum vulgare*), oat (*Avena sativa*) and Flax (*Linum usitatissimum*)^43,44^. Around 8% are used for maize (*Zea mays*) plantation for a local biogas facility^45^. (b) Cropland-type (Lower Austria- District Mistelbach): Of the 89% agricultural land of the total area of the district, around 79% are used as cropland, where predominantly various species of grain and maize (*Zea mays*) are cultivated followed by oil seed, sugar beet **(***Beta vulgaris*), wine and fruit plantation as well as fallow land; to a lower percentage, potatoes (*Solanum tuberosum*) and protein rich plants are cultivated. Only around 0.4% are defined as grassland^46^. (c) No agricultural use (Military Airport Langenlebarn, Tulln). As a control site a sampling site consisting of fallow land without any agricultural use was chosen (no land use; no fertilizer or pesticide use).

### Genomic DNA extraction

Intestinal and faecal samples were thawed on ice and total genomic DNA was extracted from 250 mg using the Powersoil^®^ DNA Isolation Kit (MoBio Laboratories Inc., Carlsbad, CA, USA) according to the manufacturer’s instructions with one modification: To ensure proper lysis of bacteria, a heating step at 70 °C for 10 min was introduced between mixing of samples with buffer C1 and bead-beating^47^. After extraction, all samples were quantified by spectrophotometry using the Qubit 2.0 Fluorometer (Thermo Fisher Scientific, Waltham, MA, USA) with the Qubit dsDNA HS Assay Kit (Thermo Fisher Scientific).

### 16S rRNA gene sequencing and sequence processing

For amplicon sequencing, we targeted the 16S rDNA hypervariable region V345, using an Illumina MiSeq sequencing platform with a 300-basepair paired-end read protocol. The universal primers 357F-HMP (5′-CCTACGGGAGGCAGCAG-3′) and 926R-HMP (5′-CCGTCAATTCMTTTRAGT-3′) were used to generate amplicons of ~570 base pairs^48,49^. Libraries were constructed by ligating sequencing adapters and indices onto purified PCR products using the Nextera XT Sample Preparation Kit (Illumina) and equimolar amounts of each of the libraries were pooled and sequenced. 16S rRNA gene PCRs, library preparation and sequencing, as well as demultiplexing and read stitching were performed by Microsynth (Microsynth AG, Balgach, Switzerland).

### Process- and sequencing- controls

Prior to sampling, DNA extraction was attempted from blank tubes and a PCR with a long 16S rRNA gene primer panel was performed to verify that sampling tubes were not contaminated. A negative control was also included in the DNA extraction run. This negative control was also sequenced and should be a proxy for reagent contamination and cross-contamination between samples. Sequencing of the negative control revealed 5,000 sequences (average read count levels per sample was 77,000). Three non-template controls and one positive control (human stool sample) were included as internal controls in library preparation. These controls were identified as expected (3 negatives, one positive).The MiSeq Run was controlled by a PhiX control sample (https://emea.illumina.com/products/by-type/sequencing-kits/cluster-gen-sequencing-reagents/phix-control-v3.html?langsel=/it).

### Microbial community analysis and predictive functional profiling

Analysis of the NGS data was conducted using the QIIME software package v1.9.1^50^. A total of 3,856,491 demultiplexed sequences were produced, of which 2,700,987 (70.04%) passed the quality filter with a phred value of >9 and a minimum read length of 520 base pairs. A total of 7,947 chimeric sequences were filtered out before operational taxonomic units (OTUs) were clustered using 97.00% identity (=0.03 distance) against the USEARCH 6.1 database^51^ and *de novo*. Singletons were removed, and representative sequences were classified via the RDP classifier 2.2^52^. Alpha diversity was calculated by subsampling the OTU table to the read depth of the sample with the lowest amount of sequences (n(seqlowest) = 8,671), resulting in 10 repetitions at 1,000 sequences up to 8,000 sequences/sample. Beta diversity was calculated by subsampling to an even read depth of 6,000 reads/sample, of which weighted UniFrac distance matrices^53^ and principal coordinate analysis were calculated^54^. Venn diagrams of OTUs were generated with the Venn diagram plotter (PNNL, Richland, WA) to display the number of OTUs shared by different populations. In addition, the core microbiota OTUs, defined as OTUs shared by over 90% of the samples, were identified. BLASTn analysis (NCBI GenBank database excluding uncultured/environmental sample sequences in the search set) of the top 50 most abundant OTUs and core microbiota OTUs were done using the highest identity matches of each unique OTUs representative sequence.

### Quantification of bacteria in the gastrointestinal tract of hares

We conducted a quantitative PCR (qPCR) to assess the absolute abundance/copy numbers of bacterial cell equivalents (BCE) present in the intestinal and faecal flora. First, quantification of all bacteria was done by targeting a conserved 16S rRNA gene region (forward primer 5′-CCTACGGGAGGCAGCAG-3′, reverse primer 5′-ATTACCGCGGCTGCTGG-3′)^55^. Second, the primer pair Eco1457F (forward primer 5′-CATTGACGTTACCCGCAGAAGAAGC-3′) and Eco1652R (reverse primer 5′-CTCTACGAGACTCAAGCTTGC-3′) were used covering a multitude of *Enterobacteriaceae*, *Erwiniaceae* and *Pectobaceriaceae*- associated genera including *Escherichia*, *Citrobacter*, *Cronobacter*, *Enterobacter*, *Shigella*, *Erwinia*, *Pantoea* and *Pectobacterium*^56^. Standards for both primer sets were made by conducting a qPCR on three pooled intestinal and faecal samples that were quantified afterwards using the Qubit Fluorometer. Copy numbers of the standard curves with genomic DNA templates were calculated with the equation: DNA (molecules/μL) = [6.02 × 10^23^ (molecules/mol) × DNA amount (g/μL)]/[DNA length (bp) × 660 (g/mol/bp)], according to Li, Penner, Hernandez-Sanabria, Oba and Guan^57^. The 16S rRNA gene copy numbers (seven copies for Eco1457F -Eco1652R qPCR, four copies for the all-bacteria targeted qPCR^58^ were taken into account when extrapolating bacterial cell equivalents (BCE).

For qPCRs, DNA samples and negative controls were run in duplicate in a 20 µl reaction. The master mix contained 10 µl 2 × Brilliant III Ultra Fast SYBR Green qPCR Master Mix (Agilent, Vienna, Austria), 2 µl of 2.5 µM primers and 5 µl of water (nuclease-free). 1 µl DNA template including approximately 10 ng DNA (intestinal samples) and 1 ng DNA (faecal samples) was added. The following amplification protocol was used: Initial denaturation at 95 °C for 3 min, 40 cycles of 95 °C for 5 s and by 20 s at 61 °C and 63 °C for general bacteria and Eco1457F -Eco1652R qPCR, respectively. A melting curve (70 °C to 90 °C, with fluorescence measurements at 1 °C intervals), was done after each qPCR and specific amplicon peaks were received. qPCR results were analysed using the Stratagene MxPro software (QPCR Software, version 2.00).

### Statistical analyses

Statistical analysis of the bacterial microbiota composition was implemented using the R statistical computing environment, version 4.3.3^59^. Alpha diversity indices were assessed by calculating the Chao1 index and the observed OTU richness. We used lme models (linear mixed-effect models, R-package, nlme^60^) to analyse the effects of independent variables, i.e. population (geographical areas (Airport (A), Lower Austria (LA) and Pellworm (P)), sex (male vs. female), age class (sub adult vs. adult), and heart fat as a parameter defining the nutritional status of the animal (ranged 1–6, whereas 1 defined cachectic, 2 bad, 3 moderate, 4 good, 5 very good and 6 described obese) on diversity indices (dependent variable). Beside these fixed effects (geographical area, sex, age, heart fat and gut health), we also used the individual animal IDs (n = 25) as a random effect. All predictor variables in the full model were checked for multicollinearity by calculating the variance inflation factor. We used the Akaike Information criteria corrected for small sample size (AICc) to determine the best model with the most relevant fixed factors for each response variable. None of the models indicated primary collinearity issues (all values were below 2.6^61^). The residuals of the models were assessed visually via histograms and Q-Q-plots as well as by calculation of the Shapiro-Wilk normality test. The dependent variable Chao1 calculated from the intestinal tract data was log transformed. The modelling procedure was carried out for both the faecal and intestinal datasets, although the effect of the faecal and intestinal samples on diversity indices itself was also calculated in an individual model run. We estimated the proportion of the variance of the fixed and random regressors using Pseudo-R-Squared (pseudo-r^2^-values^62^). In the next step, contrast coefficients of the explanatory variables were calculated among the response variables diversity indices by using a pairwise comparison of least-squares means, corrected for multiple comparison using the false discovery rate (FDR). The contrast calculation was implemented in R using the package lsmeans^63^. The level of significance was defined at *P* ≤ 0.050. The normality distribution of the abundance of phyla (n = 23), and most abundant OTUs (n = 50) were checked with the tests for multivariate data with the function manova (R Package stats) and the Cullen and Frey graph with the R package fitdistrplus and logspline^64,65^. Due to the non-normal distribution of the data, model residuals, and different identified optimal theoretical distributions of individual phyla and OTUs, the beta diversity of the microbiota of phyla and OTUs was assessed by applying a permutational multivariate analysis of variance (PERMANOVA) with the ADONIS function and 5000 permutations in R (R Package vegan^66^). To analyse whether independent variables (i.e. population, sex, age, heart fat and gut health) had also a significant effect on the composition of phyla and OTUs abundances, we calculated Bray-Curtis-dissimilarity matrix with the R function vegdist of the vegan package^66^. The distance matrix was applied as response variable. The modelling approach was carried out for faecal and intestinal samples, although the effect of the two on the composition of phyla and OTUs itself was also calculated in an individual ADONIS function. The multivariate homogeneity of group dispersions was performed with the betadisper function, a multivariate analogue of Levene’s test, followed by a permutation-based test of multivariate homogeneity of group dispersions with pairwise comparisons of group mean dispersions. The Kruskal-Wallis test was applied to identify potentially statistically significant differences between populations concerning each individual phylum, OTU, and independent variables, followed by a pairwise test for multiple comparison of mean rank sum (Dunn’s test), adjusted with Benjamini-Hochberg method (R Package PMCMR, FSA^67,68^).

Relative abundance plots of microbial communities were calculated with the transform_sample_counts function within the phyloseq package. The corresponding visualisation was conducted with the package ggplot2 in R^69,70^. The tool ‘Phylogenetic Investigation of Communities by Reconstruction of Unobserved States’ (PICRUSt) was applied to predict the metagenome functional content from amplicon sequencing^71^. Statistical analysis of metagenome predictions was carried out with STAMP^72^ by using an ANOVA with Benjamini-Hochberg FDR correction.

Additionally, the Kruskal Wallis test was applied to compare the different populations in terms of qPCR abundance of Eco1457F-Eco1652R and all bacteria in faecal samples and intestinal tract samples. A post-hoc test using Mann-Whitney tests with Bonferroni correction for multiple comparisons was used and the corresponding Z Values were computed to determine the effect size (r).

## Results

### The microbiota of intestinal and faecal hare samples

**Figure 2:**
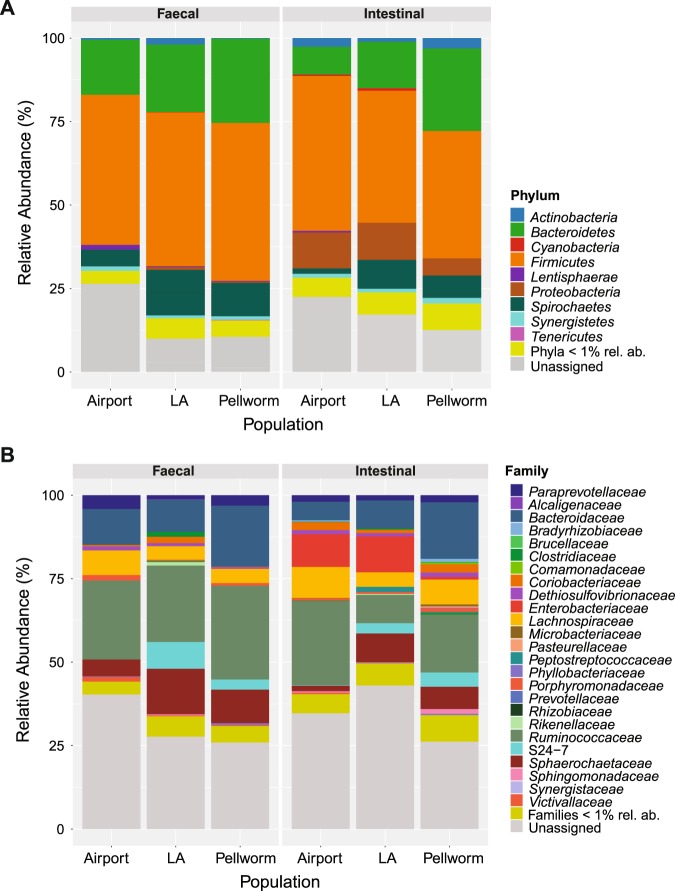
Taxonomic classification of 16S rRNA gene sequence reads parted by sampling type (i.e. intestinal vs. faecal) and population, whereas data represents average of OTU counts from replicate libraries for each category. (**A**) bacterial phyla (**B**) bacterial families in the gastrointestinal tract of hares. Phyla and families with less than 1% relative abundance were grouped together. Sequences that could not be assigned are depicted as “Unassigned”.

In total, 2,700,987 sequences (70.04%) passed our quality filter and were processed together for all downstream analysis. Reads generated for intestinal and faecal samples were distributed homogenously, with 53% reads belonging to intestinal and 47% belonging to faecal samples. At both microbiota sources analysed (intestinal and faecal samples) *Firmicutes* and *Bacteroidetes* dominated the microbial community composition (45.51% and 19.30% relative abundance over all samples respectively), followed by *Spirochaetes* and *Proteobacteria* (7.76% and 5.73% relative abundance over all samples) (Fig. 2A). In intestinal and faecal samples, 23 and 13 phyla were detected respectively. The 13 faecal-associated phyla corresponded to the highest abundance subset in intestinal samples. The phyla abundance composition differed significantly between intestinal and faecal samples (PERMANOVA: *P* = 0.002, pseudo r^2^ = 0.09; Permutest: *P* = 0.093). Intestinal samples contained an enrichment of *Proteobacteria* compared with faecal samples (15.71-fold change, *P* < 0.001) and more *Actinobacteria* (2.09-fold change, *P* = 0.002). Faecal samples contained significantly more *Lentisphaerae* (1.74-fold change, *P* = 0.050).

For intestinal and faecal samples 12,119 and 22,149 OTUs were detected over all hare populations, respectively. The overlapping pattern of abundant OTUs detected in both gut sites was high: From the 500 most abundant OTUs (89.50% of all sequences), only 1.40% (n = 7) were exclusively found in the faecal and 1.80% (n = 9) were exclusively found in intestinal samples. However, OTU abundance composition differed significantly between intestinal and faecal samples (PERMANOVA: *P* = 0.031, r^2^ = 0.04; Permutest: *P* = 0.855). In intestinal samples compared with faecal samples, a significant enrichment of OTUs with best BLAST hits to the *Escherichia-Shigella* group, *Eubacterium limosum*, *Sphingomonas kyeonggiensis*, *Flintibacter butyricus* and *Blautia faecis* were detected (Table 1). The core microbiota consisted of 66 shared OTUs, which all belonged to the 500 most abundant OTUs across all samples (Table 2).

**Table 1:** Best BLAST hits (NCBI) of the 50 most abundant OTUs.

OTU no.	best BLAST hit (NCBI)	Acc. No.	Similarity (%)	Intestinal samples (mean ab. in %)	Faecal samples (mean ab. in %)	P-Value
NR 6	*Sphaerochaeta pleomorpha*	NR_102964.1	88	5.47	9.16	n.s.
NR 0	*Sphingobacterium wenxiniae*	NR_108640.1	83	3.52	4.47	n.s.
296045	*Bacteroides sartorii*	NR_113195.1	100	2.96	3.47	n.s.
4457268	*Escherichia-Shigella* group	NR_074902.1	100	6.44	0.02	<0.001
NR 26	*Culturomica massiliensis*	NR_144745.1	87	2.62	3.53	n.s.
4303724	*Ruminococcus albus*	NR_074399.1	95	2.09	3.82	n.s.
NR 2	*Paraprevotella clara*	NR_113077.1	95	1.97	2.68	n.s.
4447072	*Bacteroides uniformis*	NR_112945.1	99	1.62	2.07	n.s.
NR 5	*Selenomonas dianae*	NR_041805.1	86	1.95	1.65	n.s.
174358	*Clostridium alkalicellulosi*	NR_115345.1	87	3.55	0.00	n.s.
215311	*Ruminococcus albus*	NR_074399.1	95	1.10	2.42	n.s.
NR 9	*Marvinbryantia formatexigens*	NR_042152.1	95	1.19	1.21	n.s.
4306729	*Ruminococcus albus*	NR_074399.1	95	0.88	1.53	n.s.
114000	*Ruminococcus albus*	NR_074399.1	95	0.75	1.48	n.s.
NR 7	*Pyramidobacter piscolens*	NR_113185.1	91	1.32	0.93	n.s.
1108377	*Muribaculum intestinale*	NR_144616.1	88	1.33	0.78	n.s.
4417325	*Bacteroides vulgatus*	NR_112946.1	99	1.09	1.01	n.s.
109753	*Pseudoflavonifractor capillosus*	NR_025670.1	93	0.27	1.76	n.s.
NR 254	*Desulfotomaculum tongense*	NR_133738.1	87	0.71	1.03	n.s.
4366089	*Flintibacter butyricus*	NR_144611.1	97	0.98	0.74	n.s.
NR 165	*Pseudoflavonifractor capillosus*	NR_025670.1	96	1.09	0.44	n.s.
NR 77	*Gracilibacter thermotolerans*	NR_115693.1	87	0.59	0.84	n.s.
289748	*Eisenbergiella massiliensis*	NR_144731.1	94	0.50	0.96	n.s.
NR 55	*Parvibacter caecicola*	NR_117374.1	91	0.91	0.54	n.s.
4468234	*Bacteroides vulgatus*	NR_074515.1	99	0.58	0.85	n.s.
4477861	*Bacteroides cellulosilyticus*	NR_112933.1	99	0.63	0.60	n.s.
NR 1	*Murimonas intestini*	NR_134772.1	95	0.78	0.53	n.s.
4461762	*Caproiciproducens galactitolivorans*	NR_145929.1	95	1.29	0.00	n.s.
NR 93	*Eubacterium limosum*	NR_113248.1	81	0.79	0.41	0.017
NR 11	*Ruminococcus albus*	NR_074399.1	94	0.38	0.78	n.s.
NR 261	*Murimonas intestini*	NR_134772.1	93	0.35	0.79	n.s.
4450360	*Sphingomonas kyeonggiensis*	NR_134182.1	100	0.72	0.09	<0.001
NR 54	*Gracilibacter thermotolerans*	NR_115693.1	87	0.42	0.61	n.s.
NR 4	*Parabacteroides johnsonii*	NR_041464.1	92	0.40	0.70	n.s.
NR 15	*Gabonibacter massiliensis*	NR_146820.1	88	0.47	0.60	n.s.
4445078	*Anaerobacterium chartisolvens*	NR_125464.1	88	0.28	0.74	n.s.
NR 43	*Oscillibacter valericigenes*	NR_074793.1	93	0.51	0.48	n.s.
4381553	*Bacteroides dorei*	NR_041351.1	99	0.42	0.58	n.s.
NR 110	*Muribaculum intestinale*	NR_144616.1	94	0.06	0.78	n.s.
2365945	*Cloacibacillus porcorum*	NR_109636.1	96	0.40	0.42	n.s.
NR 39	*Blautia hansenii*	NR_104687.1	95	0.51	0.30	n.s.
NR 198	*Thermoanaerobacter brockii*	NR_075060.1	78	0.52	0.28	n.s.
291348	*Flintibacter butyricus*	NR_144611.1	96	0.49	0.32	0.05
NR 48	*Gabonibacter massiliensis*	NR_146820.1	87	0.36	0.43	n.s.
287483	*Sphaerochaeta pleomorpha*	NR_102964.1	88	0.29	0.48	n.s.
289597	*Pseudoflavonifractor capillosus*	NR_025670.1	92	0.29	0.47	n.s.
333768	*Flavonifractor plautii*	NR_043142.1	96	0.53	0.23	n.s.
317315	*Ruminiclostridium thermocellum*	NR_074629.1	90	0.41	0.29	n.s.
NR 168	*Muribaculum intestinale*	NR_144616.1	87	0.18	0.55	n.s.
NR 57	*Blautia faecis*	NR_109014.1	94	0.43	0.21	0.047

Internal OTU numbers (no.) include New Reference (NR) OTUs from *de novo* clustering and OTUs collected against the reference collection. OTUs were blasted against NCBI GenBank nr. closest reference strains (excluding uncultured/environmental sequences). GenBank accession numbers (Acc. No.), sequence similarity, relative abundances (Mean ab. = Mean relative abundance per group) and significant abundance differences between faecal and intestinal samples are listed. Please note low sequence similarities when interpreting OTU-based data.

n.s. = not significant.

**Table 2:** The core microbiota of the intestinal tract of hares.

OTU no.	Best BLAST hit (NCBI)	Acc. No.	Similarity (%)	Intestinal samples (mean ab. in %)	Faecal samples (mean ab. in %)
NR 6	*Sphaerochaeta pleomorpha*	NR_102964.1	88	5.47	9.16
NR 0	*Sphingobacterium wenxiniae*	NR_108640.1	83	3.52	4.47
296045	*Bacteroides sartorii*	NR_113195.1	100	2.96	3.47
NR 2	*Paraprevotella clara*	NR_113077.1	95	1.97	2.68
NR 26	*Culturomica massiliensis*	NR_144745.1	87	2.62	3.53
4303724	*Ruminococcus albus*	NR_074399.1	95	2.09	3.82
NR 5	*Selenomonas dianae*	NR_041805.1	86	1.95	1.65
4447072	*Bacteroides uniformis*	NR_112945.1	99	1.62	2.07
NR 7	*Pyramidobacter piscolens*	NR_113185.1	91	1.32	0.93
NR 9	*Marvinbryantia formatexigens*	NR_042152.1	95	1.19	1.21
215311	*Ruminococcus albus*	NR_074399.1	95	1.10	2.42
NR 1	*Murimonas intestini*	NR_134772.1	95	0.78	0.53
NR 165	*Pseudoflavonifractor capillosus*	NR_025670.1	96	1.09	0.44
4417325	*Bacteroides vulgatus*	NR_112946.1	99	1.09	1.01
4366089	*Flintibacter butyricus*	NR_144611.1	97	0.98	0.74
NR 55	*Parvibacter caecicola*	NR_117374.1	91	0.91	0.54
4306729	*Ruminococcus albus*	NR_074399.1	95	0.88	1.53
NR 93	*Eubacterium limosum*	NR_113248.1	81	0.79	0.41
114000	*Ruminococcus albus*	NR_074399.1	95	0.75	1.48
NR 254	*Desulfotomaculum tongense*	NR_133738.1	87	0.71	1.03
NR 77	*Gracilibacter thermotolerans*	NR_115693.1	87	0.59	0.84
4468234	*Bacteroides vulgatus*	NR_074515.1	99	0.58	0.85
333768	*Flavonifractor plautii*	NR_043142.1	96	0.53	0.23
NR 39	*Blautia hansenii*	NR_104687.1	95	0.51	0.30
NR 43	*Oscillibacter valericigenes*	NR_074793.1	93	0.51	0.48
291348	*Flintibacter butyricus*	NR_144611.1	96	0.49	0.32
NR 15	*Gabonibacter massiliensis*	NR_146820.1	88	0.47	0.60
NR 115	*Kyrpidia tusciae*	NR_074733.1	83	0.46	0.14
NR 57	*Blautia faecis*	NR_109014.1	94	0.43	0.21
NR 54	*Gracilibacter thermotolerans*	NR_115693.1	87	0.42	0.61
4381553	*Bacteroides dorei*	NR_041351.1	99	0.42	0.58
2365945	*Cloacibacillus porcorum*	NR_109636.1	96	0.40	0.42
NR 48	*Gabonibacter massiliensis*	NR_146820.1	87	0.36	0.43
4410988	*Flintibacter butyricus*	NR_144611.1	97	0.34	0.28
305187	*Gracilibacter thermotolerans*	NR_115693.1	88	0.30	0.30
NR 156	*Murimonas intestini*	NR_134772.1	93	0.29	0.20
289597	*Pseudoflavonifractor capillosus*	NR_025670.1	92	0.29	0.47
287483	*Sphaerochaeta pleomorpha*	NR_102964.1	88	0.29	0.48
NR 84	*Ethanoligenens harbinense*	NR_074333.1	91	0.29	0.31
4328606	*Victivallis vadensis*	NR_118352.1	90	0.28	0.37
4445078	*Anaerobacterium chartisolvens*	NR_125464.1	88	0.28	0.74
109753	*Pseudoflavonifractor capillosus*	NR_025670.1	93	0.27	1.76
198331	*Gracilibacter thermotolerans*	NR_115693.1	87	0.26	0.28
NR 236	*Roseburia faecis*	NR_042832.1	92	0.26	0.18
4390211	*Bacteroides uniformis*	NR_112945.1	99	0.21	0.31
298408	*Gracilibacter thermotolerans*	NR_115693.1	87	0.19	0.18
4250634	*Bacteroides uniformis*	NR_112945.1	99	0.19	0.27
2136916	*Victivallis vadensis*	NR_118352.1	98	0.14	0.26
NR 88	*Victivallis vadensis*	NR_118352.1	97	0.14	0.26
4336940	*Clostridium sufflavum*	NR_041497.1	88	0.12	0.13
NR 24	*Cloacibacillus porcorum*	NR_109636.1	95	0.14	0.16
NR 45	*Gracilibacter thermotolerans*	NR_115693.1	86	0.12	0.13
NR 249	*Gracilibacter thermotolerans*	NR_115693.1	87	0.11	0.17
331156	*Cellulosibacter alkalithermophilus*	NR_116826.1	87	0.11	0.12
NR 154	*Roseburia hominis*	NR_074809.1	94	0.11	0.09
348839	*Desulfotomaculum tongense*	NR_133738.1	89	0.10	0.14
301464	*Gracilibacter thermotolerans*	NR_115693.1	87	0.10	0.09
4463892	*Bacteroides vulgatus*	NR_112946.1	98	0.09	0.14
NR 47	*Cellulosibacter alkalithermophilus*	NR_116826.1	86	0.08	0.08
NR 228	*Desulfotomaculum tongense*	NR_133738.1	87	0.07	0.10
297969	*Pseudoflavonifractor capillosus*	NR_025670.1	92	0.06	0.34
295527	*Gracilibacter thermotolerans*	NR_115693.1	87	0.06	0.08
4212012	*Bacteroides uniformis*	NR_112945.1	98	0.06	0.11
NR 282	*Heliophilum fasciatum*	NR_117586.1	87	0.05	0.08
NR 188	*Gracilibacter thermotolerans*	NR_115693.1	88	0.04	0.07
NR 225	*Gracilibacter thermotolerans*	NR_115693.1	88	0.02	0.04

OTUs that were present in > 90% of all samples are listed. All OTUs were additionally blasted against NCBI GenBank nr. closest reference strains (excluding uncultured/environmental sequences). GenBank accession numbers (Acc. No.) and similarity values are listed. Mean ab. = Mean relative abundance per group. Please note low sequence similarities when interpreting OTU-based data.

Observed diversity richness OTU counts and diversity estimator Chao 1 counts are listed in Supplementary Table S2. Over all populations, the average observed diversity richness per sample was 711 OTUs in faecal and 510 OTUs in intestinal samples. Chao 1 estimated the diversity on average to contain 1,686 OTUs in faecal and 932 OTUs in intestinal samples. Additionally, a statistically significant effect between intestinal and faecal abundance for the diversity indices were observed (Chao 1, *P* < 0.001 and observed OTUs *P* < 0.001). The sampling site could explain approximately 22–32% of the variance of the diversity data, but most of the variance was explained at the individual level (OTU richness: conditional r^2^ = 0.57: Chao: conditional r^2^ = 0.56).

### Factors influencing the bacterial microbiota of intestinal and faecal hare samples

The composition of the phyla abundance between different populations in intestinal samples differed by trend (PERMANOVA: *P* = 0.099, r^2^ = 0.14; Permutest: *P* = 0.148), whereas no significant effect in faecal samples could be observed (PERMANOVA: *P* = 0.610, r^2^ = 0.07; Permutest: *P* = 0.697). In particular, significant differences between different populations with respect to phyla abundance in intestinal samples were detected between *Bacteroidetes*, *Lentisphaerae* and WPS-2. *Bacteroidetes* were enriched as a trend in the Pellworm population compared with the Airport population (Airport – Pellworm, *P* = 0.054, 2.91-fold change), whereas *Lentisphaerae* and WPS-2 were significantly enriched in the Airport population (*Lentisphaerae:* Airport-LA, *P* = 0.044, 2.19-fold change; Airport-Pellworm, *P* = 0.046, 1.95-fold change; WPS-2: Airport-Pellworm, *P* = 0.049, 1.82-fold change; LA-Pellworm, *P* = 0.048, 1.70-fold change). The PERMANOVA analysis shows that age (Faecal: *P* = 0.523; Intestinal: *P* = 0.721), sex (Faecal: *P* = 0.708; Intestinal: *P* = 0.964), heart fat (Faecal: *P* = 0.482; Intestinal: *P* = 0.145) and gut health (Faecal: *P* = 0.821; Intestinal: *P* = 0.738) did not significantly affect phyla abundances. Population had further significant effects on the composition of intestinal (PERMANOVA: *P* = 0.002, r^2^ = 0.18; Permutest: *P* = 0.104) as well as faecal OTUs (PERMANOVA: *P* = 0.002, r^2^ = 0.19; Permutest: *P* = 0.258). Faecal OTUs were also significantly affected by age (PERMANOVA: *P* = 0.039, r^2^ = 0.08: Permutest: *P* = 0.441). In total, 12 and 13 OTUs from the most abundant 50 OTUs differed significantly between populations in intestinal and in faecal samples respectively: New.ReferenceOTU0 (NR0; best BLAST hit *Sphingobacterium wenxiniae*), NR11 (*Ruminococcus albus*) and NR43 (*Oscillibacter valericigenes*) were significantly enriched in the Airport population compared with the LA population (Table 3). OTU 296045 and OTU 1108377 (best BLAST hit *Bacteroides sartorii* and *Muribaculum intestinale*) had a significantly higher abundance in the Pellworm population compared with the Airport population (Table 3). NR1 (best BLAST hit *Murimonas intestine*) was higher in the Airport population compared with the Pellworm population (Table 3). Heart fat, sex and gut health parameter had no significant effect on intestinal or faecal-associated OTUs (*P* > 0.050). In this context, no significant effect of population, age, heart fat, sex and gut health on the diversity abundance in intestinal and faecal samples was observed. Our models of alpha diversity indices (Chao1 index and the observed OTU richness) could explain on average 89% of the variance of the data (conditional r^2^ = 0.88 and r² = 0.89 for intestinal and faecal samples, respectively). However, much of the variation in the data was due to the difference between individuals, since data variance explained by the fixed factors alone was considerably lower (mean marginal r^2^ = 0.08 and r² = 0.14 for intestinal and faecal samples, respectively). Abundant OTUs summed up at family level are shown in Fig. 2B.

**Table 3:** Significant OTU enrichments in intestinal and faecal samples of different hare populations.

OTU no.	best BLAST hit (NCBI)	Acc. No.	Similarity (%)	Population comparison	P-Value	fold change
**Faecal samples**						
NR 0	*Sphingobacterium wenxiniae*	NR_108640.1	83	LA- Airport	0.013	18.00
296045	*Bacteroides sartorii*	NR_113195.1	100	LA- Airport	0.027	0.09
				Pellworm- Airport	0.005	0.04
NR 26	*Culturomica massiliensis*	NR_144745.1	87	LA- Airport	0.038	3.05
NR 5	*Selenomonas dianae*	NR_041805.1	86	LA- Airport	0.002	6.58
1108377	*Muribaculum intestinale*	NR_144616.1	88	Pellworm- Airport	0.049	0.06
109753	*Pseudoflavonifractor capillosus*	NR_025670.1	93	LA- Airport	0.011	0.01
4468234	*Bacteroides vulgatus*	NR_074515.1	99	LA- Airport	0.036	3.02
				Pellworm- Airport	0.038	4.03
NR 1	*Murimonas intestini*	NR_134772.1	95	Pellworm- Airport	0.006	4.29
NR 11	*Ruminococcus albus*	NR_074399.1	94	LA- Airport	0.049	14.75
4450360	*Sphingomonas kyeonggiensis*	NR_134182.1	100	LA- Airport	0.034	0.00
NR 4	*Parabacteroides johnsonii*	NR_041464.1	92	Pellworm - LA	0.002	0.01
NR 43	*Oscillibacter valericigenes*	NR_074793.1	93	LA- Airport	0.034	5.71
				Pellworm - LA	0.041	0.33
289597	*Pseudoflavonifractor capillosus*	NR_025670.1	92	LA- Airport	0.005	0.10
				Pellworm- LA	0.052	5.24
**Intestinal samples**						
NR 0	*Sphingobacterium wenxiniae*	NR_108640.1	83	Airport - LA	0.001	0.06
296045	*Bacteroides sartorii*	NR_113195.1	100	Airport - Pellworm	0.003	25.94
4303724	*Ruminococcus albus*	NR_074399.1	95	Airport - LA	0.054	0.21
4306729	*Ruminococcus albus*	NR_074399.1	95	LA - Pellworm	0.045	17.76
1108377	*Muribaculum intestinale*	NR_144616.1	88	Airport - Pellworm	0.01	15.60
4366089	*Flintibacter butyricus*	NR_144611.1	97	Airport - LA	0.005	0.27
				Airport - Pellworm	0.044	0.37
NR 1	*Murimonas intestini*	NR_134772.1	95	Airport - LA	0.009	0.07
				Airport - Pellworm	0.006	0.14
NR 11	*Ruminococcus albus*	NR_074399.1	94	Airport - LA	0.046	0.05
NR 261	*Murimonas intestini*	NR_134772.1	93	Airport - LA	0.012	0.04
NR 43	*Oscillibacter valericigenes*	NR_074793.1	93	Airport - LA	0.002	0.14
2365945	*Cloacibacillus porcorum*	NR_109636.1	96	Airport - Pellworm	0.004	3.72
				LA - Pellworm	0.033	2.14
291348	*Flintibacter butyricus*	NR_144611.1	96	Airport - LA	0.004	0.22

OTUs were additionally blasted against NCBI GenBank nr. closest reference strains (excluding uncultured/environmental sequences). GenBank accession numbers (Acc. No.) and similarity values are listed. Only significant values are shown. Please note that low fold changes may be statistically significant, but do not represent real biological enrichments. Please also note low sequence similarities when interpreting OTU-based data.

**Figure 3:**
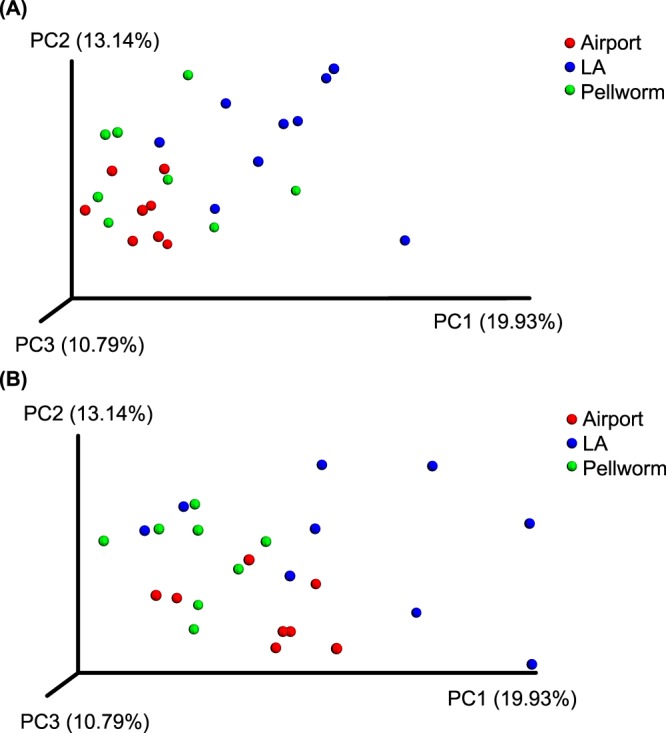
PCoA Plot based on weighted UniFrac distance matrices of faecal and intestinal samples. In Panel (**A**) faecal and in Panel (**B**) intestinal samples are depicted. UniFrac distances were calculated for all OTUs. Each point represents values from one individual with colors expressing population affiliation.

The Venn diagram (Supplementary Fig. S1) displays an unequal amount of OTUs per population and a high number of unique OTUs in each population. In faecal samples, 7.69% of all OTUs, and in intestinal samples 8.97% of all OTUs were shared between the three populations examined. The phylogenetic distance measurement done with weighted UniFrac analysis did not reveal a clear clustering of populations, however the Airport population samples were more similar to each other compared with the other populations for both intestinal and faecal samples. The highest variability within a population was found for the LA population (Fig. 3). The PICRUSt analysis indicated one significant difference on KEGG (Kyoto Encyclopedia of Genes and Genomes) Level 3 in the population comparison: The linoleic acid metabolism was significantly higher in Airport and Pellworm populations compared with LA population (Effect size: 0.36, *P* < 0.005; Supplementary Table S3).

### Bacterial cell equivalents in intestinal and faecal samples

**Figure 4:**
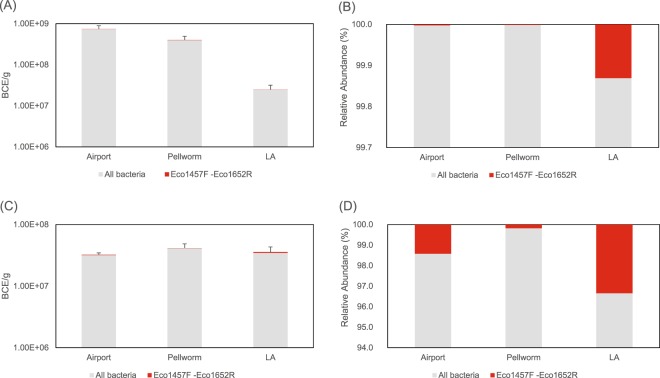
Bacterial cell equivalents (BCE) determined by qPCR. A primer panel covering all bacteria and a primer panel covering a multitude of *Enterobacteriaceae*, *Erwiniaceae* and *Pectobaceriaceae*- associated genera (Eco1457F-Eco1652R) were used for (**A**,**B**) faecal and (**C**,**D**) intestinal samples. (**A**,**C**) include absolute BCE counts, (**B**,**D**) show the relative abundance of *Enterobacteriaceae*, *Erwiniaceae* and *Pectobaceriaceae*- associated genera in proportion to all bacteria.

In 1 g intestinal samples, between 3.2E + 07 (Airport population) and 4.1E + 07 BCE (Pellworm population) were detected with qPCR. In 1 g faecal samples, between 2.5E + 07 (LA population) and 7.3E + 08 (Airport population) BCE were found. The number of *Enterobacteriaceae*, *Erwiniaceae* and *Pectobaceriaceae*, covered with the primer panel Eco1457F- Eco1652R, varied in intestinal samples between 7.4E + 04 (Pellworm population) and 1.2E + 06 (LA population), and in the faecal samples between 5.1E + 03 (Pellworm population) and 3.2E + 04 (LA population) gene copies/g (Fig. 4). In faecal samples a significant effect was identified for the factor ‘population’ on the Eco1457F- Eco1652R qPCR (χ^2^ = 11.25, *P* = 0.003). Specifically, differences were detected between the Airport and LA (*P* = 0.005, effect size r = 0.70) and between Pellworm and LA (*P* = 0.030, effect size r = 0.58).

## Discussion

This is to our knowledge the first study characterising the intestinal and faecal microbiota of the European brown hare based on 16S rRNA gene sequencing.

To date, studies describing the gastrointestinal microbiota are limited to one member of the family *Leporoidae*, the rabbit^73,74^. As sympatric relatives, often sharing the same habitat as well as feed preferences, the species do share similarities of their gastrointestinal physiology^75^. *Leporidae* are hindgut fermenters and practitioners of caecotrophy. Nevertheless, hares and rabbits show both morphological differences of their gastrointestinal tract (GIT) and different digestive strategies in terms of nutrient extraction^14,75,76^. Those differences in digestive function and strategies between the two species suggest that microbial communities in the GIT may also differ and potentially show divergent functionality or metabolic pathways. Indeed, we were able to identify that the core microbiota of the hare GIT diverges from that previously described in rabbits. Our study reveals that the gut microbiota of hares includes *Clostridium*, *Bacteroides*, and *Ruminococcus* species, but not *Streptococcus* and *Enterobacter* species as previously described to be abundant in rabbit caecum^77–82^. Despite these differences we also detected similarities between the gut microbiota of the two species, e.g. *Eubacterium* and *Ruminococcus* related OTUs, both found among the 50 most abundant and core microbiota OTUs in this study, have been described in the rabbit GIT as well^83^. Although the highly abundant phylotypes detected in this study were in accordance with phylotypes recently described in rex rabbits^74^, hare intestinal and faecal samples were highly enriched for *Spirochaetes* compared with rabbits. The majority of the phylum *Spirochaetes* consisted of the family *Sphaerochaetaceae* mainly comprising of OTUs affiliated to *Sphaerochaeta pleomorpha* or *Sphaerochaeta globosa*. These OTUs showed only 88% sequence similarity to reference 16S rRNA gene sequences but 93% to 96% sequence similarity to 16S rRNA gene sequences of uncultured *Spirochete* clones found in rumen of ruminants or the oral cavity of canines^84,85^. Interestingly, BLASTn database sequences include a large number of uncultured *Sphaerochaeta* species residing in a broad range of mammalian GIT or oral cavities. Unlike most other *Spirochaetes*, which usually have a helical morphology and flagella, *Sphaerochaeta* are spherical and not motile^86^. They are capable of heterofermentative growth on carbohydrates such as pentose and hexose monosaccharides, disaccharides and soluble starch^86^. In contrast to their closest relatives *Spirochaeta*, *Treponema*, and *Borrelia*, they are not thought to be pathogenic^87^.

Similar to the *Sphaerochaeta* associated OTUs, other high-abundance OTUs observed in this study had similarity scores to reference 16S rRNA genes below 90%, which is in accordance with a study on bacterial species in the rabbit caecum where the majority of sequences shared less than 97% identity to BLASTn database sequences^83^. Hence our data demonstrate that gastrointestinal tracts of hares harbour multiple bacterial species that that are yet to be described.

Studies in wildlife species are often restricted in sample availability due to limitations of access to free-ranging animals and restrictions in the application of invasive sampling methods. Non-invasive samples such as faeces are in many cases the only available sample rescource. In order to evalutae the representation of the gastrointestinal microbiota composition in faecal samples and therefore its potential use in future studies in hares, we tested the similarity between the faecal and intestinal microbial community composition.

*Firmicutes* and *Bacteroidetes* dominated the microbial community composition in both sample types, followed by *Proteobacteria* and *Spirochaetes*. Intestinal samples showed a higher number of phyla with a significantly different abundance pattern compared with faecal samples. Similarly, diversity indices and the abundance of OTUs differed significantly between sampling sites, indicating clear microbiota shifts along the hare’s gastrointestinal tract. Faecal samples were not representative for the description of the intestinal microbiota. This is in accordance with recent studies that identified a distinct microbial environment between luminal and mucosal sites and in respective compartments of the gastrointestinal tract of monogastric animals^88,89^. Therefore, future studies should consider using GIT samples representative for testing their specific hypothesis.

Land use practices associated with poorly diversified and restricted food availability is known to effect gut microbiota and individual fitness^11^. Highly specialized feeders, such as the brown hare, might be even more severely affected by a decrease in plant biodiversity^14^. Our three chosen sampling locations differ tremendously in land use and habitat type parameters. The agricultural land use in Pellworm consists predominantly of grassland and to a much lower percentage cropland; whereas the situation in LA is the opposite, with the majority of land used as cropland. The control site Airport consists of fallow land without any agricultural use. As highly selective feeders, hares prefer weeds and grasses, and specifically select for certain plant taxa^14^. Furthermore, they avoid crude fibre and select for high fat and energy content in their diet to provide essential polyunsaturated fatty acids (PUFA) such as linoleic acid and alpha-linolenic acid needed to reproduce and survive. A study by Popescu and colleagues found that European hares are able to selectively absorb PUFA in the gastrointestinal tract and excrete faeces that are highly depleted in PUFA and enriched in saturated fatty acids (FA)^76^. Interestingly, linoleic acid metabolism was significantly elevated in the Airport and Pellworm populations compared with the LA population (Supplementary Table S3), probably indicating the variation in food and nutrient variability between the populations in our study.

The overall phyla composition was relatively stable in hares (only intestinal samples varied between populations as a trend), but hare location had a significant impact on the OTU composition of faecal and intestinal samples between the different populations. In intestinal samples a number of specific taxa differed in abundance between the different populations. For example, there was a trend for a difference in the abundance of *Bacteroidetes* between LA and Airport and a significant difference in the abundance of OTU 296045 (best BLAST hit: *Bacteroides sartorii*), which was enriched in intestinal samples of the Pellworm population relative to the Airport population. *Bacteroides* species have been detected at high abundances in rabbit caecum in the past and are associated with healthy growth and high-weight gain^74,90^. Overall, the Pellworm population had the lowest OTU diversity. Population genetic data also suggest that Pellworm has lower diversity than the Austrian populations and its restricted geneflow is likely also associated with the low OTU diversity. Further research on associations between OTU diversity on gut health, respectively disease susceptibility and on physiological parameters is warranted. Hares of the Airport population showed lower abundances of the yet uncultured bacterial family S24-7 compared with the Pellworm and LA population. This family is being recognized as a predominant member of the intestinal tract of homeothermic animals, but data on their physiology, metabolic capacity, and interactions with the host are limited^91^. Plant glycan (hemicellulose and pectin) degradation has been described based on genomic characterization of the S24-7 family^91^. Our results suggest that both grassland and cropland agricultural areas are associated with increased abundances of S24-7 affiliated bacteria in the intestinal tracts of hares.

Population had a significant effect on both the composition of intestinal and faecal OTUs. Population was also the explanatory factor for the abundance of potential pathogenic bacteria of the family *Enterobacteriaceae*.

Many members of the family *Enterobacteriaceae*, which showed the highest abundance in the LA population and lowest abundance in hares of the Pellworm area, are connected to gastrointestinal disease^92–94^. The low abundance of *Enterobacteriaceae* and the high abundance of OTU 296045 (i.e, likely *Bacteroides*) in the GIT of Pellworm hares might indicate a healthier microbial composition in the intestinal tract. Host traits such as age, sex, body condition (heart fat) and gut health were not related to the abundance and diversity of the bacterial composition in intestinal and faecal samples of brown hares in this study.

Nevertheless, further investigations that look into associations of agricultural use, plant diversity and occurrence of dysbiosis are desirable. Considering the low number of sampled animals (n = 25) in the presented study, significant impact of independent variables should be interpreted with caution. A larger sample size in future studies would be desirable and would allow for more robust conclusions than those derived from the present study.

## Conclusion

The current knowledge of gut microbiota composition and influencing factors in wildlife species under natural conditions is extremely limited. Our aim was to provide a broad foundation to generate specific hypothesis at the intersection of gut health, land use change and population viability for the EBH and other species impacted by rapid habitat modification. This combined information is lacking to date, despite being essential for understanding gut microbial variation within wildlife species to differentiate natural variability from that related to external stressors leading to functional dysbiosis affecting host health. Anthropogenic habitat modifications together with species-specific susceptibility to these modifications might also have an impact on wildlife gut microbiota.

This study generates fundamental baseline data on the diversity and composition of gut microbiota in the brown hare that will complement previous research and create a starting point for future research to understand the physiological and pathophysiological dynamics of gut microbiota as well as the causes of alterations to the intestinal microbial communities in this species.

## Supplementary information

Supplemental Material

## Data Availability

Nucleotide sequence accession numbers: Sequencing data are available in the European Nucleotide Archive Database under the accession number PRJEB15166.
