# Supplementary material for: Gut microbiota of the European Brown Hare (Lepus europaeus)
Source: Sci Rep. 2019 Feb 25;9:2738. doi: 10.1038/s41598-019-39638-9 (PMC6390100; doi:10.1038/s41598-019-39638-9)
Supplement: Supplementary file 1 — Supplemental Material [file 41598_2019_39638_MOESM1_ESM.docx]

**Gut microbiota of the European Brown Hare (*Lepus europaeus*)**

G.L. Stalder^1*^, B. Pinior^2^, B. Zwirzitz^3,4^, I. Loncaric^5^, D. Jakupović^1^, S.G. Vetter^1^, S. Smith^6^, A. Posautz^1^, F. Hoelzl^6^, M. Wagner^3,4^, D. Hoffmann^7^, A. Kübber-Heiss^1^ and E. Mann^3^

1. Research Institute of Wildlife Ecology, Department of Integrative Biology and Evolution, University of Veterinary Medicine, Vienna, 1160 Vienna, Austria
2. Department for Farm Animals and Veterinary Public Health, Institute for Veterinary Public Health, University of Veterinary Medicine, Vienna, 1210 Vienna, Austria
3. Department for Farm Animals and Veterinary Public Health, Institute of Milk Hygiene, Milk Technology and Food Science, University of Veterinary Medicine, Vienna, 1210 Vienna, Austria
4. Austrian Competence Centre for Feed and Food Quality, Safety and Innovation FFoQSI GmbH, Technopark 1C, 3430 Tulln, Austria
5. Department of Pathobiology, Institute of Microbiology, University of Veterinary Medicine, Vienna, 1210 Vienna, Austria
6. Konrad Lorenz Institute of Ethology, Department of Integrative Biology and Evolution, University of Veterinary Medicine, Vienna, 1160 Vienna, Austria
7. Game Conservancy Deutschland e. V., Schloßstrasse 1; 86732 Oettingen, Germany

* Corresponding author:

Dr. G.L. Stalder

Department of Integrative Biology and Evolution, Research Institute of Wildlife Ecology, University of Veterinary Medicine, Vienna, Austria

Savoyenstraße 1a

A-1160 Vienna, Austria

[gabrielle.stalder@vetmeduni.ac.at](mailto:gabrielle.stalder@vetmeduni.ac.at);

Phone: +43 1 25077 7250
Fax: +43 1 25077 94 7250

**Table S1. Metadata of European brown hares included in the study.** (for detailed description of each parameter see also Material and Methods).

**Population:** The animals originated from three different geographical locations associated with different land use practices: Sampling area **(a) Pellworm**, Germany: (**Grassland type**) is characterized by 70% grassland and 30% cropland of the total agricultural area; **(b) Lower Austria**- district Mistelbach, Austria: (**Cropland-type**) 79% of the agricultural area are used as cropland; and 0.4% are defined as grassland **(c)** Military **Airport** Langenlebarn, Tulln, Austria (**No agricultural use**); **Sex:** all animals were sexed according to secondary sexual characteristics; in one animal sex was not assessed; **Age:** was determined by the weight of the dried eye lenses. Hares with an eye lens weight smaller than 276 mg were classified as subadults; **Heart fat:** defining the nutritional status ranged from 1-6, whereas 1 defined cachectic, 2 bad, 3 moderate, 4 good, 5 very good and 6 described obese; **Gut health:** was defined based on pathological examination of the GI-tract: 1: no or very mild macroscopic and/or histopathological lesions, 2: moderate macroscopic and/or histopathological lesions 3: severe macroscopic and/or histopathological lesions. For detailed description of each parameter see also Material and Methods.

|  |  |  |  |  |
| --- | --- | --- | --- | --- |
| **Population** | **Sex** | **Age** | **Heart fat** | **Gut health** |
|  |  |  |  |  |
|  |  |  |  |  |
| Pellworm | f | adult | 5 | 1 |
| Pellworm | f | subadult | 6 | 2 |
| Pellworm | f | adult | 2 | 1 |
| Pellworm | m | adult | 5 | 2 |
| Pellworm | m | adult | 4 | 1 |
| Pellworm | m | subadult | 5 | 2 |
| Pellworm | m | adult | 3 | 1 |
| Pellworm | f | subadult | 4 | 2 |
|  |  |  |  |  |
| LA | f | adult | 4 | 2 |
| LA | m | subadult | 3 | 3 |
| LA | m | subadult | 1 | 2 |
| LA | m | subadult | 3 | 2 |
| LA | f | subadult | 0 | 2 |
| LA | m | subadult | 4 | 3 |
| LA | f | subadult | 5 | 1 |
| LA | m | subadult | 3 | 1 |
| LA | m | subadult | 3 | 3 |
| Airport | f | na | 3 | 1 |
| Airport | m | subadult | 3 | 1 |
| Airport | f | adult | 4 | 3 |
| Airport | na | adult | 4 | 2 |
| Airport | m | adult | 4 | 2 |
| Airport | f | adult | 5 | 2 |
| Airport | m | adult | 5 | 1 |
| Airport | m | adult | 3 | 2 |
|  |  |  |  |  |

**Table S2.** Observed diversity richness OTU counts and diversity estimator Chao 1 counts for each population and sample type. SD= Standard deviation per population.

|  |  |  |  |  |  | |
| --- | --- | --- | --- | --- | --- | --- |
|  |  |  |  |  | |  |
|  | Population | Chao 1 estimator  (mean ± standard deviation) |  | Observed OTUs  (mean ± standard deviation) | |  |
|  |  |  |  |  | |  |
|  |  |  |  |  | |  |
|  | ***All faecal samples*** | 1685.89 ± 909.50 |  | 711.16 ± 255.89 | |  |
|  | Airport faecal samples | 2517.73 ± 1086.64 |  | 882.09 ± 309.17 | |  |
|  | LA faecal samples | 1373.87 ± 511.87 |  | 640.07 ± 150.60 | |  |
|  | Pellworm faecal samples | 646.80 ± 618.00 |  | 354.90 ± 226.43 | |  |
|  | ***All intestinal samples*** | 931.94 ± 298.10 |  | 510.27 ± 149.43 | |  |
|  | Airport intestinal samples | 878.45 ± 174.29 |  | 473.86 ± 78.53 | |  |
|  | LA intestinal samples | 1030.01 ± 396.10 |  | 536.13 ± 196.59 | |  |
|  | Pellworm intestinal samples | 875.10 ± 232.02 |  | 517.58 ± 134.18 | |  |
|  |  |  |  |  | |  |
|  |  |  |  |  |  |  |

**Table S3.** PICRUSt metagenome predictions based on 16S rRNA gene amplicons with functions being predicted against the KEGG database.
Pathways with sequence read proportions with FDR<0.15 between populations are listed.

|  |  |  |  |  |  |  |  |
| --- | --- | --- | --- | --- | --- | --- | --- |
|  |  |  |  |  |  |  |  |
| Level 2 (KEGG) | Level 3 (KEGG) | P value | FDR | Effect size | Airport: mean (%) | LA: mean (%) | Pellworm: mean (%) |
|  |  |  |  |  |  |  |  |
|  |  |  |  |  |  |  |  |
| Lipid Metabolism | Linoleic acid metabolism | <0.001 | 0.005 | 0.358 | 0.09 | 0.07 | 0.10 |
| Lipid metabolism | Unclassified | 0.012 | 0.138 | 0.172 | 0.14 | 0.13 | 0.12 |
| Lipid Metabolism | Fatty acid biosynthesis | 0.010 | 0.140 | 0.179 | 0.57 | 0.50 | 0.55 |
| Glycan Biosynthesis and Metabolism | N-Glycan biosynthesis | 0.001 | 0.089 | 0.261 | 0.01 | 0.03 | 0.02 |
| Metabolism of Cofactors and Vitamins | Riboflavin metabolism | 0.005 | 0.126 | 0.202 | 0.18 | 0.22 | 0.19 |
| Metabolism of Cofactors and Vitamins | Folate biosynthesis | 0.003 | 0.096 | 0.223 | 0.36 | 0.37 | 0.42 |
| Carbohydrate metabolism | Unclassified | 0.005 | 0.124 | 0.205 | 0.22 | 0.18 | 0.23 |
| Amino Acid Metabolism | Histidine metabolism | 0.011 | 0.137 | 0.174 | 0.67 | 0.66 | 0.73 |
|  |  |  |  |  |  |  |  |
|  |  |  |  |  |  |  |  |


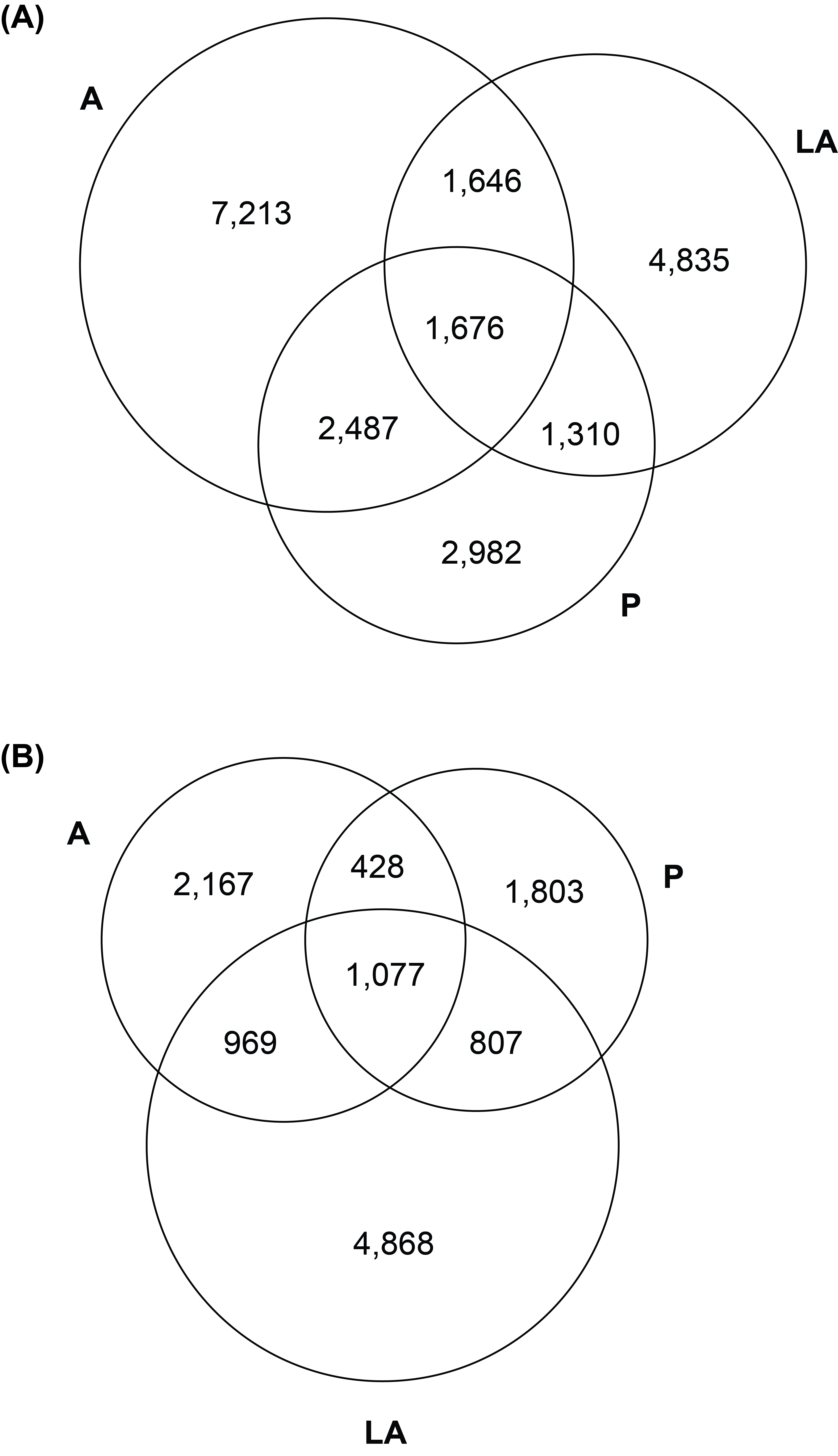


**Figure S1.** Venn diagrams with numbers of OTUs shared between the populations airport (A), Pellworm (P) and Lower Austria (LA). Circle sizes are in proportion to the number of OTUs. (A) faecal and (B) intestinal samples.
